# Using the rear-view mirror to look forward

**DOI:** 10.2807/1560-7917.ES.2023.28.2.220112e

**Published:** 2023-01-12

**Authors:** Eurosurveillance editorial team

**Affiliations:** 1 European Centre for Disease Prevention and Control (ECDC), Stockholm, Sweden

**Keywords:** COVID-19, SARS-CoV-2, metrics, impact factor, peer reviewer

As the new year begins, one cannot help but recall the start of 2020, when the severe acute respiratory syndrome coronavirus 2 (SARS-CoV-2) emerged in China and public health experts and scientists realised that a situation with possible major impact worldwide was emerging. At this time, there were many uncertainties about the virus itself, its abilities to spread and the disease it might cause. Much progress has been made since and the impact of SARS-CoV-2 and the coronavirus disease (COVID-19) is much better understood and mitigated. Irrespectively, at the start of 2023 there are new challenges. Following the lifting of the stringent COVID-19 measures (zero-COVID strategy) in September 2022 in China, the country is experiencing a massive surge of cases [1,2] while the new, highly transmissible Omicron variant sublineage XBB.1.5 continues to spread at a high pace in parts of the United States (US) [3]. Public health experts are watching and analysing the situation in both China and the US closely. Based on 564 sequences collected since 1 December 2022 from China and deposited by 3 January 2022 in the Global Initiative on Sharing All Influenza Data (GISAID) EpiCoV database, there are no clear signs of new variants circulating in the country [4]. This is in line with results from passenger screening at arrival of four different flights from China at the end of December 2022 in Italy. All 61 sequenced samples obtained among 126 SARS-CoV-2 positive passengers (total tested 556) belonged to Omicron subvariants already circulating in Europe [5]. There are no data available from the US which indicate an association of XBB.1.5 with a more severe clinical picture [3].

While COVID-19 continues to leave its mark on the world, we continue to learn and identify areas for improvement despite many lessons learnt over the past 3 years. As a scientific public health journal, *Eurosurveillance* is committed to providing a platform to share experiences and the advances made. We published, for example, several articles on integrated surveillance for respiratory diseases caused by viruses such as SARS-CoV-2 and influenza virus [6-8]. Another focus has been on structures for better preparedness and response to future health threats and crises. At the European Union level, in 2022, the Regulation on serious cross-border threats to health was adopted [9], and mandates of two EU agencies, the European Medicines Agency (EMA) and the European Centre for Disease Prevention and Control (ECDC), were expanded. Already in September 2021, the European Commission (EC) Directorate General European Health Emergency and Preparedness Authority (HERA) was established as well as the World Health Organization Hub for Pandemic and Epidemic Intelligence in Berlin, Germany. A series of editorials in our journal outlines key elements of these entities and their collaboration. Three editorials were published in 2022; two more, one by ECDC’s Director and one by the EC Directorate General Health and Food Safety (DG SANTE), will follow in the coming weeks — watch this space!

Among the 82 rapid communications that we published in 2022, there are once again several very early or even first accounts of worrisome outbreak signals such as a salmonella outbreak linked to chocolate products [10], hepatitis of unknown aetiology in young children [11] and mpox (formerly monkeypox). The mpox outbreak has been concentrated outside of previously endemic areas and predominantly affecting men who have sex with men (MSM). It was declared a Public Health Emergency of International Concern by World Health Organization on 23 August 2022 [12]. In line with our aim to provide rapid evidence for public health decision-making, we paid special attention to this epidemic which resulted in the mpox 2022 collection containing 28 articles on the topic. The outbreak has caused 25,736 cases up to 4 January 2023 in Europe [13]. It mainly affected segments of the population with established structures for risk communication and sensitised for the need of behaviour changes to prevent onward transmission and infection. The rapid decline of mpox signalled the positive impact of targeted and rapid public health measures in such a scenario.

The 10 most viewed articles in 2022 each feature aspects of SARS-CoV-2/COVID-19, mpox or hepatitis of unknown origin in young children. However, as pointed out above, we covered a range of other topics relevant for communicable diseases prevention and control. Overall, we published 216 articles (82 rapid communications, 122 regular articles and a total of 12 editorials and letters). On average, we received 81 submissions per month, slightly less than in the recent years and our acceptance rate was 20% (excluding pre-submission enquiries). Submissions were received from six continents and a total of 78 countries; while our focus is on public health in Europe, we continue to welcome submissions of articles from other parts of the world which may have an impact on public health in Europe. The metrics for the journal continue to be good. The most recent impact factor, for the year 2021, is 21 (InCites Journal Citation Reports, Clarivate analytics, 2022; rank 5/95 category Infectious Diseases journals), the highest ever obtained for the journal. The Scopus CiteScore is 22 (rank 3/562 category Medicine (Public Health, Environmental and Occupational Health); rank 4/108 Medicine (Epidemiology)), and the SCImago Journal Rank (SJR) is 45 among 2,489 journals in the category ‘Medicine miscellaneous’.

In 2022, we piloted two new initiatives: co-reviewing and a key public health message box. The co-reviewing aims to support training and capacity building of early career researchers as well as provide mentoring and guidance opportunities for the main reviewers while both receive recognition for their activity. The uptake of the pilot thus far has been somewhat slow; however, overall feedback from participants and our first impression are positive: reviews are comprehensive and there is a good focus on public health impact. We would thus like to encourage experts who receive these reviewer invitations to participate and share their experiences with us. The key public health messages that now appear at the beginning of articles are guided by three questions that aim to provide a better understanding of the content and its impact on public health for informed non-expert audiences. We will evaluate both initiatives in due time and adapt our approaches accordingly.

One increasing challenge in the past 3 years has been finding peer reviewers. Anecdotal evidence attests to a general fatigue among public health experts/scientists owed to their generally increased workload and the remarkable growth in the number of publications and the time pressure under which they are asked to operate. This makes us even more grateful to the ca 500 peer reviewers who supported us in 2022. As usual, we published a list with reviewer names as an acknowledgement of their engagement in our first issue of the new year [14]. We need the reviewers’ contributions to uphold the quality and standards of our journal, and we wholeheartedly appreciate that they dedicate extra time and efforts without remuneration to serve public health and quality assurance in scientific reporting. We are also aware of and recognise the many experts who are not named in our reviewer list but who are part of our board, colleagues at ECDC, or experts in our vast network who support us with advice and guidance time and again. Their support means a lot to us and we warmly thank all of them and we also thank our publisher, ECDC, and its Director for supporting the journal and its editorial independence.

Looking forward, what can our readers expect in 2023? We signed the Sustainable Development Goals (SDGs) publishers compact in December 2022 and as signatories we will highlight articles and initiatives supporting Goal 3: Ensure healthy lives and promote well-being for all at all ages. We will also continue to give attention to inclusive language and provide related feedback to our authors and reviewers where possible. We will gradually and increasingly include articles focussing on infectious disease drivers as well as environmental and societal factors that impact on communicable diseases and public health. First and foremost, however, we will continue to strive to select and publish relevant rapid communications and regular articles to support public health action and decision making in Europe, and we look forward to your contributions and continued support in 2023.

## References

[1] World Health Organisation (WHO). WHO meets with Chinese officials on current COVID-19 situation. Available from: https://www.who.int/news/item/30-12-2022-who-meets-with-chinese-officials-on-current-covid-19-situation

[2] European Centre for Disease Prevention and Control (ECDC). Impact of surge in China COVID-19 cases on epidemiological situation in EU/EEA. Available from: https://www.ecdc.europa.eu/en/news-events/impact-surge-china-covid-19-cases

[3] European Center for Disease Prevention and Control (ECDC). Update on SARS-CoV-2 variants: ECDC assessment of the XBB.1.5 sub-lineage. Available from: https://www.ecdc.europa.eu/en/news-events/update-sars-cov-2-variants-ecdc-assessment-xbb15-sub-lineage

[4] World Health Organisation (WHO). TAG-VE statement on the meeting of 3 January on the COVID-19 situation in China. Available from: https://www.who.int/news/item/04-01-2023-tag-ve-statement-on-the-3rd-january-meeting-on-the-covid-19-situation-in-china

[5] Novazzi F Giombini E Rueca M Baj A Fabeni L Genoni A Genomic surveillance of SARS-CoV-2 positive passengers on flights from China to Italy, December 2022. Euro Surveill. 2023;28(2):2300008. 10.2807/1560-7917.ES.2023.28.2.2300008 PMC9837854 36695479

[6] Glatman-Freedman A Gur-Arie L Sefty H Kaufman Z Bromberg M Dichtiar R The impact of SARS-CoV-2 on respiratory syndromic and sentinel surveillance in Israel, 2020: a new perspective on established systems. Euro Surveill. 2022;27(16):2100457. 10.2807/1560-7917.ES.2022.27.16.2100457 35451365 PMC9027148

[7] Boender TS Cai W Schranz M Kocher T Wagner B Ullrich A Using routine emergency department data for syndromic surveillance of acute respiratory illness, Germany, week 10 2017 until week 10 2021. Euro Surveill. 2022;27(27):2100865. 10.2807/1560-7917.ES.2022.27.27.2100865 35801521 PMC9264729

[8] Bagaria J Jansen T Marques DF Hooiveld M McMenamin J de Lusignan S I-MOVE-COVID-19 study team . Rapidly adapting primary care sentinel surveillance across seven countries in Europe for COVID-19 in the first half of 2020: strengths, challenges, and lessons learned. Euro Surveill. 2022;27(26):2100864. 10.2807/1560-7917.ES.2022.27.26.2100864 35775429 PMC9248262

[9] European Commission. Regulation (EU) 2022/2371 of the European Parliament and of the Council of 23 November 2022 on serious cross-border threats to health and repealing Decision No 1082/2013/EU. Office Journal of the European Union. Luxembourg: Publications Office of the European Union. 06.12.2022:L314/26. Available from: https://eur-lex.europa.eu/legal-content/EN/TXT/?uri=CELEX:32022R2371

[10] Larkin L Pardos de la Gandara M Hoban A Pulford C Jourdan-Da Silva N de Valk H Investigation of an international outbreak of multidrug-resistant monophasic *Salmonella* Typhimurium associated with chocolate products, EU/EEA and United Kingdom, February to April 2022. Euro Surveill. 2022;27(15):2200314. 10.2807/1560-7917.ES.2022.27.15.2200314 35426359 PMC9012091

[11] Marsh K Tayler R Pollock L Roy K Lakha F Ho A Investigation into cases of hepatitis of unknown aetiology among young children, Scotland, 1 January 2022 to 12 April 2022. Euro Surveill. 2022;27(15):2200318. 10.2807/1560-7917.ES.2022.27.15.2200318 35426362 PMC9012090

[12] World Health Organization (WHO). Second meeting of the International Health Regulations (2005) (IHR) Emergency Committee regarding the multi-country outbreak of monkeypox. Geneva: WHO; 2022. Accessed: Available from: https://www.who.int/news/item/23-07-2022-second-meeting-of-the-international-health-regulations-(2005)-(ihr)-emergency-committee-regarding-the-multi-country-outbreak-of-monkeypox

[13] European Centre for Disease Prevention and Control/WHO Regional Office for Europe. (ECDC.WHO/Europe). Joint ECDC-WHO Regional Office for Europe Mpox Surveillance Bulletin. Stockholm: ECDC; 4 Jan 2023. Available from: https://monkeypoxreport.ecdc.europa.eu/

[14] Eurosurveillance editorial team. Eurosurveillance reviewers in 2023. Available from https://www.eurosurveillance.org/content/10.2807/1560-7917.ES.2023.28.1.2301051.

